# Clinically accountable school health: a case for extending primary care into schools

**DOI:** 10.3389/fpubh.2026.1922178

**Published:** 2026-09-14

**Authors:** John Mugisha, Julius Niyibizi, Dexing Zhang

**Affiliations:** 1 Rwanda Implementation Science and Health Systems Lab (RISHS Lab), Kigali, Rwanda; 2 School of Nursing, Faculty of Health and Social Services, The Hong Kong Polytechnic University, Hong Kong, Hong Kong SAR, China; 3 Jockey Club School of Public Health and Primary Care, The Chinese University of Hong Kong, Hong Kong, Hong Kong SAR, China

**Keywords:** child and adolescent health, health systems strengthening, health workforce, primary health care, Rwanda, school health, School Health Officer

## Abstract

Rwanda’s primary and secondary schools serve about 4.7 million learners, creating an important platform for child and adolescent health. Rwanda has school-health policies, referral arrangements, and strong primary health care (PHC) and community systems, but it lacks a standardized, funded, and clinically regulated function consistently responsible for school-linked first response, referral coordination, and follow-up. This Perspective argues that this continuity gap at the school PHC interface deserves explicit attention and proposes a School Health Officer (SHO) as one model to test within existing PHC structures. We outline a bounded SHO scope, map the mechanisms through which the role could contribute to the six WHO health system building blocks, and identify safeguards related to workforce capacity, financing, data governance and role clarity. Rather than assuming national scale-up, we propose a risk-stratified pilot that compares feasible delivery configurations and assesses access, referral completion, workload, equity, safety and cost. The purpose is to determine whether a PHC-linked SHO function adds value to existing school-, community- and facility-linked arrangements before wider adoption is considered.

## Introduction

Rwanda’s schools serve about 4.7 million primary and secondary learners (1). This figure demonstrates the reach of the school platform, not the prevalence of illness among learners. Even so, Rwanda-specific evidence documents health needs that intersect with schooling, including menstrual-health barriers, mental-health needs among adolescents, epilepsy and disability-related challenges (2–8). These data do not provide a single estimate of clinical events occurring during school hours, but they show why schools are relevant settings for early recognition, safe initial response, referral, and follow-up.

Rwanda has also invested substantially in PHC, community health, and health-workforce development (9–12), alongside a longstanding school-health policy architecture. The National School Health Strategic Plan assigned responsibilities across education and health actors, including school-health committees, focal teachers, referral arrangements, and participation of a nurse from the nearest health facility. WHO’s review of Rwanda’s school-health services similarly noted that policy envisioned periodic nurse visits from primary care, but implementation was not consistently evident (13, 14). The gap is therefore not an absence of policy, goodwill, or activity. The unresolved question is whether existing arrangements provide sufficiently clear and continuous clinical accountability from first response through referral and follow-up at the school PHC interface.

To inform this Perspective, we searched PubMed, Scopus, Web of Science, and Google for literature and policy documents relevant to school health in Rwanda. Searches focused on school health in Rwanda and related issues, including child and adolescent health, primary care, community health workers, referral, governance, information systems, and financing. We reviewed identified sources manually and prioritized Rwanda-specific research and policy documents, supplemented by WHO guidance and relevant evidence from other LMICs. We included sources when they directly informed the argument or proposed SHO model. The search informed the Perspective and was not intended to constitute a systematic review.

## The gap is governance, not goodwill

Rwanda’s current school health arrangements depend on multiple actors, each with an important but partial role. Teachers observe learners daily and often provide first-line support. Health facilities diagnose and treat illness. Parents and caregivers remain central to decision-making and follow-up. However, when multiple actors share responsibility without a designated, trained, and supervised clinical lead, the response to a learner in need becomes uncertain.

Rwanda-specific evidence identifies opportunities to strengthen links between schools, community health workers (CHWs) and PHC (15). At the same time, studies from Rwanda and other LMICs document substantial CHW workload and resource constraints (16–20). These findings do not establish that CHWs cannot contribute to school health; rather, they caution against assigning additional clinical functions without defining scope, supervision, time requirements, and workload implications. Teachers likewise remain central to recognition, first aid, communication, and referral, but their role differs from that of a clinically regulated professional. The appropriate balance among teacher-, CHW-, facility- and SHO-linked approaches should therefore be tested rather than assumed.

The equity implications are important. Menstruation-related absenteeism remains a significant barrier to girls’ education in sub-Saharan Africa, and in Rwanda, with inadequate facilities, menstrual pain, and stigma contributing to missed school and widening educational inequalities (2–5). Learners with epilepsy, asthma, diabetes, disability, sensory impairment, or mental health needs should not have their safety determined by school resources, geography, or staff confidence (6, 7). The SHO should identify repeated health-related absences, coordinate access to menstrual supplies, screen for disability-related barriers, and ensure that learners with chronic conditions are referred and followed up.

## A proposed School Health Officer function within primary care

We propose that Rwanda test an SHO function: a clinically qualified, professionally regulated role embedded within PHC and linked to schools. The design premise is not to medicalize schools, but to create a clearer clinical interface between schools and the health system. Clinical supervision, professional regulation and performance management would remain within the health sector, while the education sector and school leadership would remain essential partners in access, coordination, safeguarding and implementation. This co-governed arrangement should be evaluated, not assumed to be superior to existing or alternative models.

At a minimum, a nurse or clinical officer could deliver the function within existing professional regulation and training pathways. Schools would host or facilitate the service, while clinical decisions and supervision would remain within established health-sector structures. The purpose would be to test whether a named PHC-linked clinician improves continuity and accountability without creating an unnecessary parallel workforce or displacing essential facility-based services.

The proposed function has three core domains. First, the SHO would provide immediate responses during school hours. This includes triage, first aid, recognition of danger signs, seizure safety, acute distress support, injury management, safeguarding escalation, and communication with caregivers and facilities. Second, the SHO would support planned prevention and care coordination. This includes protocolized screening or screening coordination for vision, hearing, dental health, nutrition-related risks, disability-related needs, menstrual health concerns, and early mental health warning signs, as well as care plans and school accommodations for learners with chronic conditions. Rwanda’s national mental health survey documented substantial unmet need and low service utilization (8).

Schools are an important point for early identification and referral, though not a substitute for specialist care. Third, the SHO would support continuity of care and appropriate data visibility. Referral follow-up should aim to establish whether the learner reached care and whether school-based support needs to change. Routine service data could include a limited set of encounters, emergencies, screenings, referrals and follow-up indicators. Any reporting into HMIS or linkage with EMIS should use a minimum approved dataset, comply with Rwanda’s data-protection requirements, and avoid unnecessary duplication of identifiable clinical information across systems.

## What the SHO addresses across Rwanda’s health system

The value of the SHO is a hypothesis about how a PHC-linked clinical function might address system-level problems, not an observed result. Table 1 therefore maps the main system issues the proposal aims to address and the mechanisms to test in a pilot.

**Table 1:** Conceptual mapping of proposed SHO mechanisms across the WHO health system building blocks.

WHO building block	Current gap	Proposed mechanism to test
Service delivery	Teachers, families, CHWs, and health facilities address school health needs, but continuity between first response, referral, and follow-up may vary.	Test whether a named PHC-linked contact improves safe first response, referral initiation, and follow-up within a defined scope.
Health workforce	Rwanda does not currently have a standardized clinically regulated school-based role spanning the functions proposed here.	Test whether a designated nurse/clinical officer function improves coordination without inappropriate task expansion or displacement of facility/CHW work.
Information systems	School-linked clinical encounters and referrals are not consistently visible in routine health-system reporting.	Define and test a minimum approved indicator set and referral/follow-up reporting pathway without duplicating unnecessary identifiable clinical records across HMIS and EMIS.
Medicines and technologies	Availability, resupply and oversight of first-response commodities may vary between schools.	Test a standardized school-health kit and resupply process linked to existing PHC supply and supervisory arrangements.
Financing	The cost and feasible staffing configuration for an SHO model are not known.	Estimate personnel time, training, supervision, commodities, data and referral costs across alternative delivery configurations.
Leadership and governance	Policy assigns school-health responsibilities across sectors, but operational clinical accountability at the school PHC interface may remain diffuse.	Test explicit roles for health and education actors, professional supervision, escalation pathways and accountability for referral follow-up.

## Implementation: from proposal to pilot: what should be tested

Three issues should be treated as pilot questions rather than assumptions: workforce feasibility, role boundaries, and affordability. Rwanda’s 4 × 4 Health Workforce Reform provides a policy context for workforce expansion (9, 21), but it does not establish that an SHO model is affordable or that existing staff can be redeployed without opportunity costs. A pilot should therefore compare feasible delivery configurations, such as a dedicated school-based function in selected high-need settings, cluster-based coverage and facility-linked outreach. The purpose of comparison would be to identify which configuration, if any, offers an acceptable balance of access, continuity, safety, workforce demand, equity and cost.

The model should also avoid unnecessary medicalization. The SHO scope would remain limited to protocolized first response, screening or screening coordination, referral initiation and follow-up, chronic-condition care planning, safeguarding escalation and selected health-promotion support. We propose that diagnosis, prescribing and definitive treatment remain within existing authorized health services rather than becoming routine SHO functions. CHWs and teachers would remain important partners; the pilot should clarify where their roles complement the SHO and where additional task transfer would be inappropriate.

Risk stratification is useful only if the criteria are defined before implementation. Candidate criteria could include school size, boarding status, distance from a health facility, inclusive-education needs, health-related absenteeism or documented referral burden. A future pilot protocol should predefine the selection criteria, the unit of implementation or comparison (for example, school or school cluster), the comparison model or usual arrangement, the main outcomes, their denominators and data sources, and how important baseline differences between schools will be handled. This Perspective does not claim that the SHO is effective or cost-effective; those are empirical questions for the pilot.

Core evaluation outcomes should include referral completion and the timeliness and appropriateness of responses to predefined school health events. Additional outcomes should cover access and equity, learner and caregiver experience, safeguarding processes, SHO/teacher/CHW workload, displacement of facility services, and data completeness and safety. The economic component should estimate personnel time, training, supervision, commodities, transport/referral costs, data-system costs, and coverage per SHO or delivery unit. These parameters would allow later estimation of cost per learner served and, if suitable outcome data are available, cost-effectiveness. The pilot aims to generate context-specific evidence for a later policy decision, not to confirm a predetermined model.

## What we are calling for

The Ministries of Health and Education should jointly commission a time-bound, adequately funded pilot in selected districts representing different implementation contexts. The pilot should be embedded within existing PHC and education structures and use predefined criteria for feasibility, safety, equity, workload, and value. Consider wider adoption only if those criteria are met, and the model offers an advantage over strengthened existing arrangements or other delivery configurations.

Governance should be shared, but roles should be explicit. Professional registration, clinical supervision, and clinical performance management would sit within the health sector, while the education sector would support school access, coordination with school leadership, and alignment with education policy. If the pilot is promising, financing options should then be assessed within routine PHC planning and national budget processes rather than assuming a dedicated financing mechanism in advance.

## Risks and safeguards

The proposed function could fail if its role is vague, trained staff is diverted into school administration, supervision is weak, or school health kits are not reliably supplied. These are not secondary concerns; they are core design requirements.

Several safeguards are therefore essential. The SHO scope should remain limited to first response, screening or screening coordination, referral initiation and follow-up, chronic-condition care planning, safeguarding escalation, selected health promotion support, adolescent-friendly support, and routine data reporting. Health facilities should remain responsible for diagnosis, prescribing, and definitive treatment, in line with national regulations. Professional registration should provide accountability and legal protection. Commodity performance should be reviewed as part of routine PHC health management processes. Selection, training, and supervision should explicitly assess confidentiality, non-judgmental adolescent care, disability inclusion, and protection from stigma. Without trust, earned through confidentiality, respect, and consistent presence, a school health service may exist in form while failing in function.

Information governance should be treated as a core design requirement because the proposed service would handle children’s health information. Rwanda’s Law No. 058/2021 relating to the protection of personal data and privacy classifies health status and medical records as sensitive personal data. Before introducing any HMIS-EMIS linkage, the pilot should define the minimum data required, the lawful basis for processing, the responsibilities of data controllers and processors, role-based access, retention and security requirements, and procedures for responding to breaches. Identifiable clinical records should not be duplicated in EMIS simply because technical linkage is possible; where education-sector reporting is useful, aggregated or otherwise appropriately minimized indicators should be preferred unless individual-level sharing is necessary, lawful and explicitly governed. Under the proposed model, identifiable clinical records generated through the SHO would remain under health-sector governance and be accessible only to appropriately authorized personnel, while education-sector access would be limited to the minimum information required for agreed educational, safeguarding, or referral functions.

## Discussion and conclusion

Rwanda’s schools already participate in health promotion, first aid, referral and links with families, CHWs and health facilities. The case for an SHO therefore does not rest on the claim that school health is absent. It rests on a narrower question: whether a named, clinically regulated PHC-linked function could improve continuity across recognition, first response, referral and follow-up in ways that current arrangements do not consistently provide.

The SHO should consequently be treated as a model to test rather than a solution already validated. Its value would depend on whether it improves referral completion, safety and equity without creating excessive workforce demands, duplicating existing roles, weakening facility services or introducing unaffordable costs. The same pilot should allow comparison with strengthened teacher-, CHW- or facility-linked approaches so that the policy decision is about the best way to organize school-PHC accountability, not simply whether to adopt a new job title.

A carefully specified pilot would also allow Rwanda to resolve questions that this Perspective cannot answer from the available literature: which schools should receive more intensive support, which delivery configuration is workable, what minimum data should be collected, how clinical and education information should be governed, and what level of investment produces sufficient value. These are empirical questions and should remain open until tested.

If the pilot demonstrates acceptable feasibility, safety, equity and value, Rwanda could consider phased expansion within existing PHC planning and workforce reforms. Experience from such a pilot may also inform other LMICs seeking stronger school-health-system links, but transferability should be treated as a hypothesis requiring adaptation to local policy, workforce, financing, and data-governance contexts rather than as an established property of the SHO model.

## Data Availability

The original contributions presented in the study are included in the article/supplementary material, further inquiries can be directed to the corresponding author/s.
